# Implementing safe abortion in Ghana: “We must tell our story and tell it well”

**DOI:** 10.1002/ijgo.12674

**Published:** 2018-10-30

**Authors:** Wendy Chavkin, Peter Baffoe, Koku Awoonor‐Williams

**Affiliations:** ^1^ Mailman School of Public Health and Department of Obstetrics‐Gynecology Columbia University Medical Center New York NY USA; ^2^ Global Doctors for Choice New York NY USA Accra Ghana; ^3^ Obstetrics and Gynecology Ghana College of Physicians and Surgeons Accra Ghana; ^4^ Policy Planning Monitoring and Evaluation Division Ghana Health Services Accra Ghana

**Keywords:** Advocacy, Ghana, Implementation, Legalization, Midlevel provider, Public health, Safe abortion, Task sharing

## Abstract

In the first years of the new century, the Ministry of Health/Ghana Health Service determined to reduce abortion‐associated morbidity and mortality by increasing access to safe care. This was accomplished by interpreting Ghana's restrictive law so that more women qualified for legal services; by framing this effort in public health terms; by bundling abortion together with contraception and postabortion care in a comprehensive package of services; and by training new cadres of health workers to provide manual vacuum aspiration and medical abortion. The Ministry of Health/Ghana Health Service convened medical and midwifery societies, nongovernmental organizations, and bilateral agencies to implement this plan, while retaining the leadership role. However, because of provider shortages, aggravated by conscientious objection, and because many still do not understand when abortion can be legally provided, some women still resort to unsafe care. Nonetheless, Ghana provides an example of the critical role of political will in redressing harms from unsafe abortion.

## Abstract 2

Starting in 1994, Ghana reinterpreted its abortion law to broaden access and reduce unsafe provision. Implementation of services has been aided by task sharing, multisector cooperation, and political will.

## METHODOLOGY FOR ALL CASE STUDIES

This case study is one of six comprising a comparative examination of varied countries’ approaches to the implementation of national abortion service programs, after changes in laws or policy guidelines that established or expanded access to services. In addition to Ghana, case studies were conducted in Colombia, Ethiopia, Portugal, South Africa, and Uruguay, as they had all either implemented new abortion laws or policies, or changed interpretations of existing laws or policies, within the past 15 years. Each study used the Integrated Promoting Action on Research Implementation in Health Services (i‐PARIHS) framework to organize the analyses. i‐PARIHS posits successful implementation to be a function of the *innovation* to be implemented and its intended *recipients* in their specific context, with *facilitation* as the “active ingredient” aligning innovation and recipient.^1^ For each country case, two types of data sources were used: an in‐depth desk review and 8–13 semistructured, in‐depth interviews with key stakeholders and experts in each country, selected in collaboration with in‐country partners. Respondents provided written informed consent and were guaranteed confidentiality. Several respondents from each country served as in‐country coauthors, in doing so giving up their anonymity as participants of the study, although no quotations provided as respondents are directly attributed to them. Respondents included healthcare providers, public health and government officials who had been involved in establishing or expanding the service, academics, and members of nongovernmental organizations (NGOs) and legal and feminist advocacy groups; in some countries interviewees came from the full range listed, in others, from a subset (Table 1). Interviews were conducted in English by a physician member of the team. Quotes presented are from interviews without attribution as we promised confidentiality. Data analysis comprised a multistep iterative thematic analysis, with coding structured to follow the i‐PARIHS framework. The Ghana Health Service Ethical Review Committee approved the case study conducted in Ghana. A full discussion of methodology can be found in Chavkin et al.^2^

**Table 1:** Professional domains of interviewees in Ghana

Professional domain	Number of interviewees
Medical practitioners	3
Government officials	3
NGO staff	1
Other^a^	1

Abbreviation: NGO, nongovernmental organization.

a “Other” comprises academics, or individuals from feminist or legal advocacy groups, or UN agencies.

## 1 CONTEXT

Ghana is considered one of Sub‐Saharan Africa's most stable democracies. Nonetheless, it is still a lower‐middle‐income country, with concerning health outcomes and shortages of practitioners and medical supplies. There are disparities between the urban centers and the rural areas, with the latter experiencing more severely limited resources and worse health outcomes.^3^ Recognizing that these inequalities had been aggravated in preceding decades by the imposition of user fees, the Ministry of Health and its implementing arms, Ghana Health Service (GHS) and Health Facilities Regulatory Agency, have attempted a variety of efforts to expand medical care for the population, and aspire to universal health coverage. In 2003, Ghana became one of few Sub‐Saharan African countries to establish a national health insurance program—the NHIS—which relies on public financing and user premiums, although vulnerable groups are generally exempted from the latter, and receive free but limited care.^3^, ^4^ Many services are not funded, and because the public sector is underfinanced, private spending on health is among the highest in Africa.^3^ The public system is partially supported by international aid, NGOs, and the Christian Health Association of Ghana (CHAG), and private practice physicians are significant care providers.^3^ The Ministry of Health is tasked with ensuring that each subgroup is aligned with national policies and standards although its surveillance and enforcement capacities are limited.

Reproductive health outcomes are among those of concern in Ghana. Contraceptive prevalence is low and unmet need for contraception and maternal mortality are both high (roughly 30% and 319 per 100 000 live births, respectively).^5^ Abortion is still an offence under Ghana's Criminal Code, although since 1985 exceptions have been available in cases of rape, incest, serious fetal anomaly, or if the woman's health is at risk.^6^ Yet neither the general public nor clinicians appeared to clearly understand that abortion is legally permitted in these circumstances. Many women therefore turned to unqualified providers and received unsafe procedures, with associated high rates of death and morbidity.^7^, ^8^ With 22%–30% of maternal deaths in Ghana thought to be attributable to unsafe abortion, the Ministry of Health and GHS have considered unsafe abortion to be an important, remediable public health issue.^9^

Therefore, in 1994, the Ministry of Health/GHS expanded its efforts to prevent unsafe abortion to include postabortion care (PAC), which could be provided by midwives as well as physicians.^10^, ^11^, ^12^

In 2006, the Ministry of Health/GHS convened NGOs, UN agencies, and medical societies to develop comprehensive abortion care guidelines and to “facilitate the provision of a package of services called Comprehensive Abortion Care that will reduce unwanted pregnancy and abortion‐related maternal morbidity and mortality”.^5^, ^6^

## 2 INNOVATION

The Ministry of Health utilized its standard mechanism for developing policies and protocols. At its behest, the GHS convened a working group of key stakeholders, including NGOs, development and donor bilateral partners, international agencies (including UNFPA and WHO) and medical societies to develop policies and guidelines for the provision of comprehensive abortion care. The framework for promoting comprehensive abortion care in Ghana was a public health one, specifically to reduce maternal deaths. While the colonial era law remains unchanged, the key innovation in Ghana was to “relax the law,” that is, to interpret the law so as to maximize access to safe abortion services. This framing was widely embraced. One interviewee reported:

…a public health argument and a medical argument were the main arguments. So first postabortion care, then comprehensive abortion care…to reduce morbidity and mortality…the first thing we went to was the need to reduce maternal mortality and that was the core message.

The law permits abortion on grounds of “foetal impairment, rape, incest, physical and mental health, life, and defilement of a female idiot,” if performed by a registered medical practitioner in an approved facility.^6^ The comprehensive abortion care guidelines include broad interpretation of the category of “maternal health” as a ground for legally permissible abortion. Requirements for qualifying under the other permissible categories were “relaxed”: women qualifying for mental health reasons do not need psychiatric assessment, minors do not need to obtain parental consent, and rape victims do not have to report to the police. The guidelines stipulate that options counseling must be provided to those considering abortion and that family planning must be offered at the time of the procedure.^13^

In 1994, when the GHS embarked on the provision of postabortion care to remedy the morbidity and mortality associated with unsafe procedures, midwives were trained to use manual vacuum aspiration (MVA).^10^ As the law permits abortion provision by any registered medical practitioner, the comprehensive abortion care guidelines specify that midwives can provide first‐trimester abortion procedures using medication abortion as well as MVA; the protocols have since been revised accordingly and mifepristone has been registered in the country. An interviewee explained:

What worked well is load sharing, task sharing, so at first, we used to have a lot [of] women who come to the gyne department, maybe with a retained placenta or a criminal abortion, or had taken something, we have to wait for the doctors to finish what they are doing. So, this woman can be there for more than 24 hours. But when midwives are trained, they are always around, they could act rapid, they are the first people to see these women. And if they're trained, they're going to reduce maternal mortality and morbidity…

## 3 RECIPIENTS

The Ministry of Health/GHS convened the NGOs who had been important reproductive service providers and champions; the UN agencies and donors whose technical expertise, funding, and credibility were highly valued; and the national medical and midwifery/nursing societies, whose members constitute the professional healthcare workforce.

NGOs such as Global Doctors for Choice, Ipas, Marie Stopes International, Pathfinder, and Venture Strategies Innovations provided abortion care, clinical training for the various cadres involved in provision, advocacy training and values clarification, and sometimes direct funding. The WHO provided protocols, including for task sharing, and supported capacity building, and UNFPA provided funding for implementation, equipment, and monitoring; sometimes NGOs supplemented the UNFPA contributions with their own staff and financial resources.

Support from the professional societies was essential and forthcoming. As most physicians are generalists, the Ghana Medical Association's endorsement was needed as was that of the Society of Gynaecologists and Obstetricians of Ghana.

The two sectors of physicians that proved harder to integrate into comprehensive abortion care were the private physicians, who do not work for the GHS, and the physicians who work for CHAG and contract with the GHS. While both groups are supposed to be subject to Ministry of Health policies, oversight and enforcement are limited. One interviewee said:

I mean private is private. They are regulated by certain rules and guidelines but …whatever is good for their benefit, they take it from the Ministry. Whatever is not [good] for them they leave it. So, you have a private facility, they have to use the new policy guidelines. But if they use it or not, nobody is going to follow up.

Expanding the role and skills of midwives was a key component of the new effort. The Nursing and Midwifery Council was supportive but had two concerns: that this expanded job capacity be adequately recognized or compensated, and that they maintain regulatory authority over their members. An interviewee explained:

There was thinking that gradually doctors are shifting their responsibilities to nurses and midwives, and the nurses and midwives are not getting extra recognition or extra remuneration for this. Also, these midwives would….show proficiency [and] were going to be in a higher grade, level…there were some fears, we found out later through the grapevine, that the council was worried they were going to lose control of those categories of person, and that those were going to be passed to the Medical Council [and Dental] for regulation, instead of nurses.

No monetary supplements are offered to clinicians who provide abortions in public sector facilities; midwives obtain certification and CME credit for undergoing training and “top‐up” trainings and have opportunities to participate in desired training retreats and workshops. One interviewee reported:

Ipas has a quarterly kind of retreat for everybody, they all come, we go out of town, we have a retreat for like 2–3 days, stay in a hotel… So that was a big event for them…

The GHS together with the NGOs considered it essential to gain support from certain groups not traditionally included in public health efforts, but whose roles could obstruct or support safe abortion. Because of the persistence of widespread misunderstanding of the law and the circumstances in which comprehensive abortion care can be legally provided, they worked to inform the police and judges about the new policies, as they might otherwise prosecute women for obtaining abortions.^8^ One interviewee said:

You need to work with everybody. We work with the police. Because if they say ‘oh this girl was raped and take her to the police station’ and this girl is pregnant, and they would say ‘no because you were raped you cannot have an abortion’ but when they understood it, the ones we worked with, they would rather call us and say ‘so and so, we have somebody, where should we send this person?’ So, the key people should understand the importance of that law that allows some people, to have termination done.

Women are the ultimate recipients of the service and there have been direct educational efforts to the public, which have depended on print and radio. Another innovative approach has been to engage and inform local community leaders: the “Queen Mothers.” One interviewee reported:

When we invited them [Queen Mothers] for this, they came, we gave them a lot of [information] so they go back into their communities, and then they have a day, for their ladies and the young ones, they tell them we don't want you to be getting pregnant without wanting it. And if it should happen, there are resources, you can go here, you can go there.

According to interviewees, these efforts have had an impact but need to be sustained and funding for them has proven erratic.

## 4 FACILITATION

Presenting comprehensive abortion care as a response to a public health problem was considered a strategic success. This framing apparently helped to defuse opposition as it shifted the focus to serious death and morbidity resulting from unsafe procedures. One interviewee elaborated:

We gather them around to tell them about the program, give them the statistics about women who are dying needlessly, for them to understand that what we have come to do is not a position from America but something that Ghanaians needed to do. We're trying to help save lives. So the whole facility will be aware. We go from the head of the hospital to the janitor, for everyone to understand, everybody who is on the facility, this facility provides such services.

The leadership role of the GHS was considered essential in the rollout of comprehensive abortion care services. Despite some episodic tensions between some of the participant organizations, interviewees reported that the collaboration functioned well and supported the leadership and authority of the GHS. Respondents from NGOs, as well as from GHS, concurred that the GHS had to be responsible for ensuring that services are provided and aligned with the new standards. While all agreed that NGOs had made critically important contributions such as providing funds and clinical care, training midlevel providers to use MVA and providing the MVA kits, and holding values clarification sessions for clinical staff, they also expressed concern about reliance on NGOs as the GHS has to be able to sustain the service. Not only is programmatic involvement by NGOs often geographically and time limited, but there have also been occasional tensions between NGOs around turf, and episodes of community backlash when an NGO had proceeded too aggressively, without adequate attention to local context. One interviewee recounted:

We had follow‐up trainings, but not regularly because of funding challenges. We are unable to have regular trainings, but with the support of our partners, they provide the funding. But the unfortunate thing is that they usually do not provide to all regions. They have selected regions, so if we are going to do follow‐up training then, it's those who are not funded by an NGO or any partners.

The professional societies have supported the incorporation of relevant material into the curricula for medical and midwifery students, have participated in certifying competence, and recognized that task sharing is necessary to enable sufficient personnel to provide abortion services, particularly in rural areas. An interviewee explained:

For health officers, you have to have your logbook, you're supposed to have done some number of abortions before your OB‐GYN rotation will be signed off. Their certification regulation is based on existing practices. So, if you are a midwife, there are certain skills you are supposed to provide.

Monitoring and evaluation of the expanded services has enabled the program to improve. The GHS monitors clinical and process parameters for all services, including comprehensive abortion review. Review of these data enabled the GHS to identify trouble spots and revise program design accordingly. Additionally, several NGOs embedded research components into their programs. One interviewee said:

We [regional GHS] used to have the maternal mortality summit twice a year; we bring people from other regions. And deliberately, if there was an abortion case, or we look for some near‐miss cases due to abortion, and we do special presentations for them, how those could have been avoided.

While the data are limited, surveys report an increase in women's knowledge of the law from 4% in 2007 to 11% in 2014, and an increase in women's self‐reported capacity to obtain an abortion from 12% to 25%.^14^, ^15^

## 5 REMAINING CONCERNS

There is no organized antiabortion presence in Ghana. However, many in Ghana are very religious and believe that their religious teachings oppose abortion.^12^ Religiously based opposition has led to high levels of stigma toward both women seeking the service and clinicians providing it. This is sometimes expressed as conscientious objection by doctors and midwives who refuse to provide legal abortions. Respondents in this study concurred that these refusers do generally refer patients to those willing to perform the abortion, including those working at CHAG facilities. This aligns with findings from an earlier study on conscientious objection in northern Ghana that even objectors thought that the GHS should regulate and constrain conscientious objection.^16^ An interviewee reported:

Initially because the stigma related to abortion, midwives who were even willing and eligible to be retrained had to be persuaded by their spouses and their friends not to. But people were getting to know that they were saving lives. And they are seeing the results. So, a lot of midwives are now even knocking on our doors to be trained. Initially it was quite difficult to get eligible and willing providers.

As previously mentioned, low levels of both patient and provider familiarity with the law and the availability of legal comprehensive abortion care prevail. While we described earlier some creative efforts to inform and engage law enforcement and community leaders, there are insufficient funds to broaden these informational campaigns. One interviewee said:

I always say that [education campaigns are] the Achilles’ heel of the Ghana program. Because truly, for all the things that we have in the law, in the guidelines, trying to protect women, the population do not know. In fact, even the service providers when we started our advocacy, the service providers themselves weren't sure.

Provider shortage is pervasive in Ghana. Although this is not officially sanctioned, some localities permit fee sharing as an incentive to encourage abortion provision; however, there is a significant consequence for access in these facilities, as this imposes costs on patients. An interviewee explained:

As part of incentive for them to provide a service, there was an agreement that clients should pay for a service charge, like co‐pay, and a percentage should be given to the providers to motivate them to provide… it goes as much as 100 Cedis, which is about US $35…, GHS initially put a cap on it of about between 40 and 60 Cedis. But the midwives go as much as 100 Cedis. But let me say that when a client comes and does not have money, is not turned away. Service is provided.

Several emphasized the importance of training and working with private physicians and bringing their clinical practice in conformity with standards, but acknowledged the dearth of funds to do so. One interviewee stated:

They are the ones who are using the D&C, and they do most of the abortions. Because people who need to do an elective abortion, they go to a quiet place, not to a big hospital where everyone is there. And so, there's a need to invest in training them, and advocate for them to change the mode of practice.

The pervasive constraint is the inadequate level of resources and funding available. There are too few clinicians, rural areas of high need, and too few funds for ongoing training and education of the population.

## 6 LESSONS LEARNED

Ghana is an exemplar of the critical role of political will in redressing harms from unsafe abortion, even without changing the colonial era criminal code. The governmental health sector's assumption of responsibility and oversight was key to successful implementation of abortion services and provided coherence to the contributions of the other stakeholders. Framing abortion as a public health measure intended to reduce morbidity and mortality and locating it within a comprehensive service package also proved highly strategic and minimized opposition. Task sharing is essential in a low‐resource provider shortage setting like Ghana's; recommendations to facilitate it are to provide recognition and incentives for those acquiring new skills and responsibilities, and to reassure the various professions that they will not lose turf or authority. Lastly, data must be collected in a way to enable analysis of clinical and operational patterns and problems for ongoing assessment and improvement. The interviewees in Ghana described constraints and problems while expressing pride in the approach and program. As they said, “we must tell our story and tell it well” so that others can learn from this effort.

## AUTHOR CONTRIBUTIONS

WC: Developed initial proposal and interview instrument, conducted Ghana interviews, wrote first draft of paper, and collated edits and reviews. PB: Interviewee, served as in‐country point person, provided information and details while writing, and reviewed, corrected, and edited manuscript at multiple points. KA‐W: Advised on interview instrument, interviewee, served as in‐country point person, provided information and details while writing, and reviewed, corrected, and edited manuscript at multiple points.

## CONFLICTS OF INTEREST

PB and KAW functioned as key informants, were interviewed, and served as coauthors. The authors have no conflicts of interest to declare.

## References

[1] Harvey G , Kitson A . PARIHS revisited: From heuristic to integrated framework for the successful implementation of knowledge into practice. Implement Sci. 2016;11:33. 27013464 10.1186/s13012-016-0398-2 PMC4807546

[2] Chavkin W , Stifani BM , Bridgman‐Packer D , Greenberg JMS , Favier M . Implementing and expanding safe abortion care: An international comparative case study of six countries. Int J Gynecol Obstet. 2018;143(Suppl.4):3–11. 10.1002/ijgo.12671 30374987

[3] Saleh K . The Health Sector in Ghana: A Comprehensive Assessment. New York: The World Bank; 2012.

[4] Agyepong IA , Adjei S . Public social policy development and implementation: A case study of the Ghana National Health Insurance scheme. Health Policy Plan. 2008;23:150–160. 18245803 10.1093/heapol/czn002

[5] Sedgh G . Abortion in Ghana. New York: Guttmacher Institute; 2010. 20653094

[6] Government of the Republic of Ghana . Law 102: The Criminal Code (Amendment) Law. The Constitution of Ghana: Ghana Publishing Corporation, Accra and Tema; 1985.

[7] Morhee R , Morhee E . Overview of the law and availability of abortion services in Ghana. Ghana Med J. 2006;40:80–86. 17299572 10.4314/gmj.v40i3.55256 PMC1790853

[8] March N , Aniteye P , Mayhew S . Attitudes and experiences of women admitted to hospital with abortion complications in Ghana. Afr J Reprod Health. 2011;15:47–55. 21987937

[9] Aniteye P , Mayhew SH . Shaping legal abortion provision in Ghana: Using policy theory to understand provider‐related obstacles to policy implementation. Health Res Policy Syst. 2013;11:23. 23829555 10.1186/1478-4505-11-23 PMC3708749

[10] Rominski SD , Lori JR . Abortion care in Ghana: A critical review of the literature. Afr J Reprod Health. 2014;18:17–35. PMC4465587 25438507

[11] Odoi‐Agyarko H . Profile of Reproductive Health Situation in Ghana. 2003 http://www.who.int/countries/gha/publications/Reproductive_Health_Profile.pdf. Accessed August 14, 2018.

[12] Aboagye PK , Gebreselassie H , Asare GQ , Mitchell EMH , Addy J . An Assessment of the Readiness to Offer Contraceptives and Comprehensive Abortion Care in the Greater Accra, Eastern and Ashanti Regions of Ghana. Chapel Hill: Ipas; 2007.

[13] Ministry of Health of the Republic of Ghana . Prevention & Management of Unsafe Abortion: Comprehensive Abortion Care Services Standards and Protocols. Accra, Ghana: Ministry of Health; 2012.

[14] Ghana Statistical Service (GSS), Ghana Health Service (GHS), and ICF International . Ghana Maternal Health Survey 2014. Rockville, USA: GSS, GHS, and ICF; 2015.

[15] Ghana Statistical Service (GSS), Ghana Health Service (GHS), and Macro International . Ghana Maternal Health Survey 2007. Calverton, MD: GSS, GHS, and Macro International; 2009.

[16] Awoonor‐Williams JK , Baffoe P , Ayivor PK , Fofie C , Desai S , Chavkin W . Prevalence of conscientious objection to legal abortion among clinicians in northern Ghana. Int J Gynecol Obstet. 2018;140:31–36. 10.1002/ijgo.12328 28940197

